# Ethnic disparities in the clustering of risk factors for cardiovascular disease among the Kazakh, Uygur, Mongolian and Han populations of Xinjiang: a cross-sectional study

**DOI:** 10.1186/1471-2458-12-499

**Published:** 2012-07-03

**Authors:** Nanfang Li, Hongmei Wang, Zhitao Yan, Xiaoguang Yao, Jing Hong, Ling Zhou

**Affiliations:** 1 The Center for Hypertension of the People's Hospital of Xinjiang Uygur Autonomous Region, The Center for Diagnosis, Treatment and Research of Hypertension in Xinjiang, Urumqi, China

**Keywords:** Clustering, Cardiovascular disease risk factors, Ethnicity, Prevention

## Abstract

**Background:**

Chinese Uygur, Kazakh, Mongolian and Han populations represent >90% of the total population of Xinjiang Uygur Autonomous Region, and their genetic backgrounds, customs, culture, and food consumption are different. The effect of ethnic differences on cardiovascular disease risk factors (CRFs; hypertension, obesity, diabetes, dyslipidemia, smoking) can be striking but is rarely studied. We report here the findings of the relationship among these ethnic groups and their CRFs across the four largest ethnic groups of Xinjiang.

**Methods:**

A cross-sectional survey of representative samples was conducted 2002–2008 in Chinese Uygur, Kazakh, Mongolian and Han populations (age >30 years; 4,421 Kazakh, 3884 Han, 3,218 Uygur, and 892 Mongolian individuals) in Xinjiang.

**Results:**

A total of 90.4% of Kazakh, 91.9% of Uygur, 90.4% of Mongolian, 85.1% of Han individuals had at least one CRF. Clustering of ≥2 or ≥3 of these risk factors was noted in 65.2% or 32.1% of Kazakh, 64.8% or 33.0% of Uygur, 66.9% or 36.5% of Mongolian as well as 62.0% or 28.3% of Han subjects, respectively. Compared with the Han population, the adjusted odds ratios of ≥1, ≥2, and ≥3 CRFs for Kazakh, Uygur and Mongolian populations were higher (all P<0.001). The age-standardized prevalence of the clustering of ≥1, ≥2, and ≥3 CRFs in Kazakh, Uygur, Mongolian, and Han populations was lower than their counterparts in the NHANES Ш study (USA) but higher than in the InterASIA Study (China).

**Conclusions:**

Ethnic groups living in Xinjiang had striking differences in CRFs. Ethnic-specific strategies should be developed to prevent cardiovascular disease in different ethnic groups.

## Background

Cardiovascular disease (CVD) is a major public health problem in “developed” and “developing” countries. It results in an enormous economic burden to the societies in these types of countries [1,2]. Hypertension, obesity, smoking, dyslipidemia, and diabetes mellitus (DM) are other common cardiovascular disease risk factors (CRFs) and have a causal role in the pathogenesis of CVD [3,4].

Studies have shown that CRFs tend to cluster, and that the risk for CVD increases substantially with each additional risk factor [5]. The incidence of CRFs in developing countries is believed to be low. However, recent evidence suggests that the prevalence of CRFs has increased in economically developing countries whereas it has decreased in economically developed countries [6-8]. Moreover, the prevalence of CVD and CRFs varies by region. For example, in China, the incidence and mortality rates for CVD have been noted to be higher in northern regions compared with those in southern regions [9,10]. Furthermore, Gu et al. found that, compared with southern regions, the prevalence of CRFs (including hypertension, obesity, current smoking, dyslipidemia and DM) was higher in northern regions [11].

The Xinjiang Uygur Autonomous Region is located in northern China and is at a high altitude. Four major ethnic groups, Chinese Uygur, Kazakh, Mongolian and Han, represent >90% of the total population of Xinjiang (2007 Annual Review of Xinjiang). Their genetic backgrounds, customs, culture, and food consumption are different. It is well known that hypertension, obesity, DM and dyslipidemia are multifactorial disorders influenced by genetic and environmental exposure. Studies have found striking differences across ethnic groups with respect to the prevalence of CVD and CRFs in other parts of the world [12,13]. However, little is known about the prevalence of CRFs among the four ethnic groups in Xinjiang. It is not known whether there are differences in this prevalence between this region and other regions in China, or in developed countries such as the USA. Furthermore, in providing a context for policy planners and health education programs for different ethnicities, it is important to quantify the proportion of the population at high risk for CVD in different ethnic groups. Such data may provide valuable information for the understanding of the unique prevalence of risk factors among different ethnic populations. Hence, special preventative strategies and interventions can be developed to lower the incidence of CVD in different ethnic groups.

The present study was designed to evaluate ethnic differences in the clustering of CRFs across the Chinese Uygur, Kazakh, Mongolian and Han populations of Xinjiang. It was also designed to quantify the proportion of people who had one or more of certain CRFs (hypertension, dyslipidemia, DM, current smoking, being overweight) across these four ethnic groups. In addition, the prevalence of having ≥1, ≥2, and ≥3 CRFs in the study was compared with the Chinese population in general as well as in the International Collaborative Study of Cardiovascular Disease in ASIA (InterASIA Study) [11] and the USA population in the Third National Health and Nutrition Examination Survey (NHANES Ш ) [14].

## Methods

### Study population

The study was approved by the Ethics Committee of the People’s Hospital of Xinjiang (Xinjiang, China). Written informed consent was obtained from all subjects before data collection and measurements.

A three-stage stratified sampling method was used to select representative samples of subjects from the Kazakh, Uygur, Mongolian, and Han communities in the Xinjiang Uygur Autonomous Region of China. In the first stage, Xijiang was stratified into north and south, as delineated by the Tianshan Mountain. Three counties were randomly selected from each region: Fuyun, Fukang, and Hefeng in the northern region and Yutian, Luofu, and Hetian in the southern region. Areas that were as populated or more populated than the county’s capital were classified as “urban areas”, and towns that were less densely populated than the capital served as “rural areas”. In the second stage, one street district or township was selected randomly from each urban and rural area. In the third stage, individual participants (who were aged >30 years) were selected for inclusion in all selected areas. Only one participant was selected from each family.

A total of 15,785 persons were selected randomly from the 12 primary sampling units and invited to participate. A total of 13,356 individuals completed the survey and examination (84.6%). The overall response was similar among men and women and in urban and rural areas.

### Data collection

A set of standardized questionnaires was completed. The questions were based on: demographics; self-reported history of stroke, myocardial infarction, and congestive heart failure; previous diagnosis and treatment of hypertension, obesity, high blood levels of cholesterol, DM and other diseases; family history of hypertension, DM and stroke; drug treatment, obesity, or being overweight; education; alcohol consumption; and cigarette smoking. Cigarette smoking was defined as having smoked at least one cigarette per day for one or more years during the participant’s lifetime. Data collection was conducted in examination centers at local health clinics in the participant’s residential area. For a few individuals, the interview and examination were conducted during a home visit. Data collection was undertaken by trained and certified physicians who could speak Kazakh, Uygur, Mongolian and Han languages.

For each participant, blood-pressure measurements were obtained by a standardized protocol adapted from procedures recommended by the American Heart Association. Body weight and height were measured by trained observers following a standard protocol. Hypertension was defined as a mean systolic blood pressure (SBP) ≥140 mmHg and/or a mean diastolic blood pressure (DBP) ≥90 mmHg and/or self-reported current treatment for hypertension with antihypertensive medication. The body mass index (BMI) was calculated using the following equation:

(1) $$\text{BMI }=\text{ body weight }\left(\text{kg}\right)/{\left(\text{height }\left(\text{m}\right)\right)}^{2}$$

Being overweight was defined as having a BMI ≥24 kg/m^2^.

### Laboratory measurements

Venous blood was drawn from all participants who had fasted for ≥12 h. Serum was separated immediately and stored at −80°C. All blood samples were examined within 1 month in the Clinical Center of the People’s Hospital of Xinjiang. Serum levels of total cholesterol (TC), high-density lipoprotein-cholesterol (HDL-c), low-density lipoprotein-cholesterol (LDL-c), triglycerides (TGs) and fasting blood glucose (FBG) were obtained by enzymatic methods. Dyslipidemia was defined as self-reported current treatment with cholesterol-lowering medication or having one or more of the following serum levels: TC ≥5.18 mmol/L, TGs ≥1.7 mmol/L, HDL-c <1.04 mmol/L, or LDL-c ≥3.37 mmol/L. DM was defined as having a fasting plasma glucose level ≥7.0 mmol/L and/or self-reported current treatment of DM.

### Statistical analyses

All analyses were restricted to participants without a history of myocardial infarction, stroke, and congestive heart failure, and were standardized to the age distribution for participants. Participants were also excluded if they had missing measurements with respect to: height; weight; serum levels of TC, HDL-c, TG, or LDL-c; FBG; or blood pressure. This left a final study population of 12,415 subjects. The breakdown of the population was 4,421 Kazakh, 3,884 Han, 3,218 Uygur, and 892 Mongolian individuals.

Data analyses were undertaken using SPSS ver16 (SPSS, Chicago, IL, USA). The distribution of clinical characteristics among participants stratified by ethnic groups was analyzed using one-way ANOVA or the chi-square test. The age-standardized prevalence of the population with ≥1, ≥2, and ≥3 risk factors of CVD was determined by ethnic group separately, and the significance of the differences across subgroups was compared with the chi-square test. The adjusted odds ratios (ORs) and 95% confidence intervals (CIs) of having ≥1, ≥2, and ≥3 major risk factors for CVD compared with having no risk factors for CVD were determined from multivariate logistic-regression models that included age, sex, and ethnicity. After standardization to the age distribution by the direct method for Chinese populations from the 2000 Census, the prevalence of having ≥1, ≥2, and ≥3 CRFs in the Kazakh, Uygur, Mongolian and Han populations in Xinjiang was compared with the general Chinese population in the InterASIA Study and the USA population in the NHANES Ш study by the Wald *χ*^*2*^ test.

## Results

### Clinical characteristics

Table 1 details the clinical characteristics of the study participants. SBP, DBP, as well as levels of TC and LDL-c, were significantly higher for Kazakh and Mongolian populations than for Uygur and Han populations (all *P*<0.001). FBG, the BMI, and the prevalence of dyslipidemia and being overweight were significantly higher for Kazakh, Uygur and Mongolian populations than for the Han population (all *P*<0.001). TG levels were significantly higher for Uygur and Han populations than for Kazakh and Mongolian populations (*P*<0.001). HDL-c levels were significantly lower for the Uygur population than for Kazakh, Mongolian and Han populations (*P*<0.001). Drinking and smoking status was significantly lower for Uygur and Kazakh populations than for Mongolian and Han populations (all *P*<0.001). The prevalence of DM was significantly higher for the Uygur population than for Han, Kazakh and Mongolian populations (*P*<0.001).

**Table 1:** The clinical characteristics among participants in XinJiang stratified by ethnic groups

	**Kazakh**	**Uygur**	**Mongolian**	**Han**	***P***
	**n=4421**	**n=3218**	**n=892**	**n=3884**	
Age (y)	46.7±11.58	51.4±13.41	47.7±11.07	45.4±12.50	<0.001
Gender (male, %)	42.4	41.9	38.0	50.2	<0.001
SBP (mmHg) *	139.1±28.13	130.0±26.04	137.6±28.44	118.9±19.27	<0.001
DBP (mmHg) *	86.9±15.24	79.4±15.00	88.2±16.52	77.4±11.23	<0.001
BMI (kg/m2) **	26.3±4.56	26.6±4.46	26.5±4.57	24.5±3.62	<0.001
TC (mmol/L) *	4.77±1.16	4.41±1.30	4.84±1.14	4.70±1.14	<0.001
TG (mmol/L) #	1.19±0.82	1.56±1.26	1.15±0.91	1.47±1.23	<0.001
LDL-c (mmol/L) *	2.97±0.99	2.48±1.12	3.06±1.17	2.50±1.13	<0.001
HDL-c (mmol/L) §	1.22±0.44	1.18±0.46	1.39±0.57	1.59±0.56	<0.001
FBG (mmol/L) **	5.45±1.35	5.54±2.44	5.73±2.45	5.25±1.99	<0.001
Smoking (%) &	16.6	15.7	27.5	36.1	<0.001
Drinking (%) &	13.7	10.6	24.0	38.3	<0.001
Overweight (%) **	66.0	70.0	67.5	52.5	<0.001
Dyslipidemia (%) *	68.6	72.1	63.1	49.0	<0.001
Diabetes (%) ##	7.3	19.6	7.5	9.1	<0.001

SBP; systolic blood pressure, DBP; diastolic blood pressure, BMI; body mass index, TC; serum total cholesterol, TG; triglyceride, LDL-c; low-density lipoprotein cholesterol, HDL-c; high-density lipoprotein cholesterol, FBG; fasting blood glucose, 2HPG; 2-hour postprandial glucose.

The differences between ethnic groups were performed by *F* test or by *χ*^*2*^ test.

*; significantly higher for Kazakh and Mongolian than Uygur and Han.

**; significantly higher for Kazakh, Uygur and Mongolian than Han.

#; significantly higher for Uygur and Han than Kazakh and Mongolian.

§; significantly lower for Uygur than Kazakh, Mongolian and Han.

&; significantly lower for Uygur and Kazakh than Mongolian and Han.

##; significantly higher for Uygur than Han, Kazakh and Mongolian.

### Age-standardized prevalence of the clustering of ≥1, ≥2, and ≥3 CRFs among Kazakh, Uygur, Mongolian and Han populations

**Figure 1:**
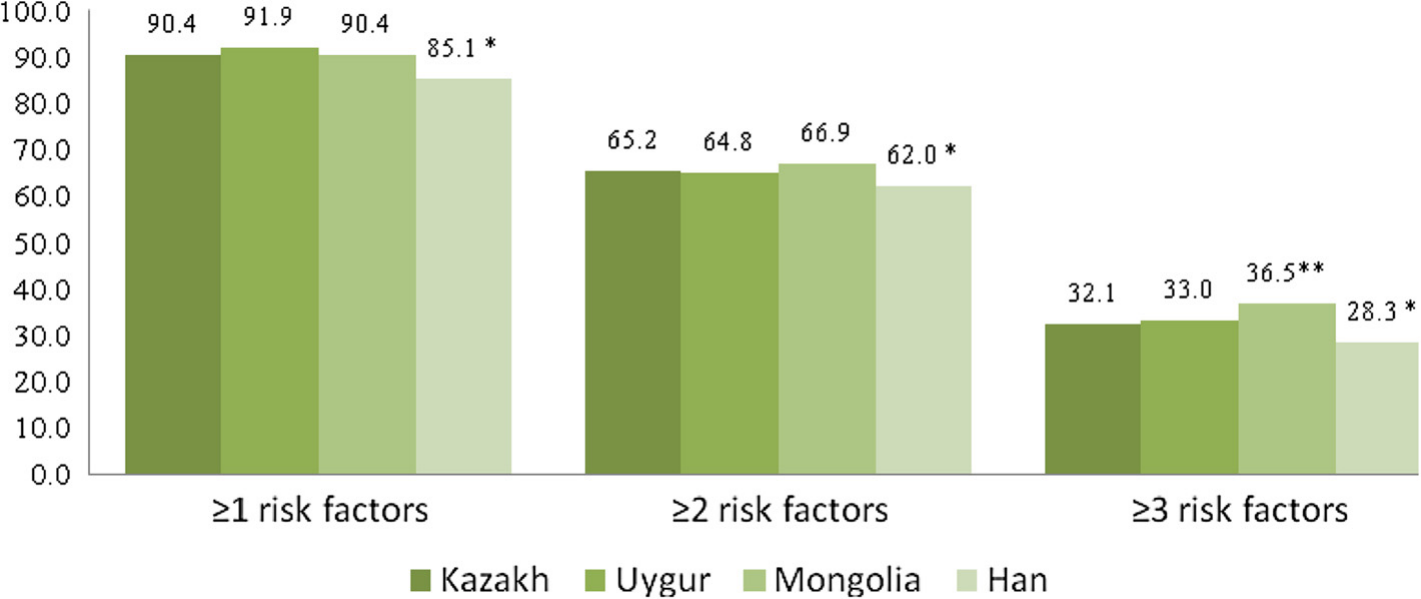
**Age-standardized prevalence of ≥1, ≥2, ≥3 cardiovascular disease risk factors among participants in Xinjiang stratified by ethnic groups (%).** * Significant lower than other populaton (*P*<0.001). ** Significant higher than other populaton (*P*<0.001).

In total, 90.4%, 65.2% and 32.1% of Kazakh, 91.9%, 64.8% and 33.0% of Uygur, 90.4%, 66.9% and 36.5% of Mongolian, and 85.1%, 62.0% and 28.3% of Han populations had clustering of ≥1, ≥2, and ≥3 CRFs, respectively (Figure 1). The age-standardized prevalence of the clustering of ≥1, ≥2, and ≥3 CRFs were significantly lower in the Han population than in the Kazakh, Uygur and Mongolian populations.

Compared with the Han population, the age- and sex-adjusted ORs of ≥1, ≥2, and ≥3 CRFs for the Kazakh population were 1.987, 2.373, and 2.283; for the Uygur population were 2.212, 2.860, and 2.440; for the Mongolian population were 2.152, 2.653, and 2.892, respectively (Table 2).

**Table 2:** **Adjusted odds ratios ( 95% confidence intervals ) of having ≥1, ≥2, ≥3*Vs*. none Cardiovascular disease risk factors associated with ethnic group**

	**≥1 Risk Factor**	**≥2 Risk Factors**	**≥3 Risk Factors**
Ethnic group			
Han	1.00 (ref )	1.00 (ref )	1.00 (ref )
Kazakh	1.987 (1.734-2.277)	2.373 (2.049-2.747)	2.283(1.926-2.705)
Uygur	2.212 (1.883-2.598)	2.860(2.411-3.393)	2.440 (2.006-2.968)
Mongolian	2.152(1.672-2.769)	2.653 (2.037-3.457)	2.892 (2.152-3.887)

The odds ratios for ethnic grouping are adjusted for age and gender.

### Prevalence of the clustering of CRFs: comparison with the InterASIA Study (China) and NHANES III Study (USA)

The age-standardized prevalence of ≥1, ≥2, and ≥3 CRFs in Kazakh, Uygur, Mongolian, and Han populations was lower than their counterparts in the USA but higher than those in the InterASIA Study (Table 3).

**Table 3:** Age-Standardized prevalence of ≥1, ≥2, ≥3 cardiovascular risk factors clustering among Kazakh, Uygur, Mongolian, Han, Inter ASIA Study (China), and United States (%)

**Groups**	**Cardiovascular risk factors clustering**		
	**≥1**	**≥2**	**≥3**
Xinjiang of China			
Kazakh	89.3	62.7	30.4
Uygur	91.6	67.8	31.4
Mongolian	89.1	64.5	34.6
Han	83.5	57.0	26.4
Inter ASIA Study			
China	80.5	45.9	17.2
NHANES III			
United States	93.1	73.0	35.9

Standardized on the basis of the year 2000 age distribution of the Chinese population.

## Discussion

The results of the present study indicated that 90.4% of Kazakh, 91.9% of Uygur, 90.4% of Mongolian, 85.1% of Han individuals had at least one of the following CRFs: hypertension; dyslipidemia; DM; were current smokers; and were overweight. Clustering of ≥2 or ≥3 of these CRFs was noted in 65.2% or 32.1% of Kazakh, 64.8% or 33.0% of Uygur, 66.9% or 36.5% of Mongolian, and 62.0% or 28.3% of Han subjects, respectively. Compared with the Han population, the adjusted ORs of ≥1, ≥2, and ≥3 CVD risk factors for Kazakh, Uygur and Mongolian subjects were higher.

Several studies have noted the striking differences across ethnic groups in the prevalence of CRFs and CVD in other parts of the world [12,13]. A key finding from the current study was that the Chinese Kazakh, Uygur, Mongolian and Han populations had distinct CRF clustering. The age-standardized prevalence of the clustering of CRFs was higher in Uygur, Kazakh and Mongolian populations than in the Han population even though the current smoking status was higher in the Han population. The significantly higher prevalence of ≥1, ≥2, and ≥3 CRFs in Uygur, Kazakh and Mongolian populations compared with the Han population may be due to the striking differences across ethnic groups with regard to the prevalence of hypertension, DM, dyslipidemia, obesity and being overweight. After a careful re-analysis of the characteristics of participants from the populations in the present study, it appears that the Kazakh, Uygur and Mongolian populations had a higher prevalence of hypertension (53.9%, 40.0%, and 55.9%, respectively), being overweight (66.0%, 70.0%, and 67.5%, respectively) and dyslipidemia (68.6%, 72.1%, and 63.1%, respectively) compared with the Han population (hypertension, 33.0%; being overweight, 52.5%; dyslipidemia, 49.0%). The striking differences across ethnic groups with regard to the prevalence of CRFs suggests the need for the development of ethnic-specific and cost-effective CVD prevention programs and health services to reduce the prevalence of CRFs as well as morbidity and mortality from CVD in the Chinese Uygur, Kazakh, Mongolian and Han populations in the Xinjiang in China.

With regard to the striking differences across ethnic groups in the prevalence of hypertension, DM, dyslipidemia, obesity and being overweight, the mechanisms underlying this phenomenon are not clear. It is believed that different environmental exposures among Chinese Uygur, Kazakh, Mongolian, and Han ethnic groups may play an important part. Besides the Han population, the inhabited area of Chinese Uygur, Kazakh and Mongolian populations is relatively isolated and fixed. Most Kazakhs and Mongolians live as herders and reside in the villages and forests north of Xinjiang, which are cold and semi-arid, whereas most Uygurs live as farmers in the plains south of Xinjiang, which are hot and arid. Moreover, Chinese Uygur, Kazakh and Mongolian share similar dietary habits. These are characterized by drinking strong wine, eating more animal fat, with a higher salt intake (>20 g per day) and consuming less grain, fresh vegetables, beans, bean products, and unsaturated fatty acids [15]. In addition to different environmental exposures among Chinese Uygur, Kazakh, Mongolian, and Han ethnic groups, differences in genetic backgrounds and gene–environment interactions could also be important factors underlying the different prevalence of hypertension [16-19]. A further study between these CRFs and ethnic-specific genetic susceptibility is needed to clarify this observation.

There is emerging evidence that the synergistic effect of CRFs clustering is associated with CVD and a higher prevalence of cardiovascular events [20-23]. Recent studies have confirmed that the clustering of these risk factors has more harmful cardiovascular effects than that predicted by a single risk factor [21-23]. In those studies, CVD incidence and all-cause mortality increased substantially in the presence of progressively more risk factors. For example, using data from the First NHANES Epidemiologic Follow-up Study, the age-, race-, sex-, and education-adjusted relative risks of coronary heart disease during 21 years of follow-up in adults with 1, 2, 3, 4 or 5 CRFs (hypertension, high cholesterol, DM, being overweight, and current smoking) compared with their counterparts with none of these CRFs were 1.6, 2.2, 3.1, and 5.0, respectively [23]. In the present study, the much higher age-standardized prevalence of having clustering of ≥1, ≥2, and ≥3 CRFs (hypertension, dyslipidemia, DM, current smoking, and being overweight) were detected in Chinese Kazakh (90.4%, 65.2% and 32.1%, respectively), Chinese Uygur (91.9%, 64.8% and 33.0%, respectively) and Mongolian (90.4%, 66.9% and 36.5%, respectively) subjects, which suggests that those people were exposed to a higher risk of CVD. Clearly, more effective prevention efforts targeting CRFs are needed in these populations. Also, future public health interventions need to take into account the special needs of people living in Xinjiang. Another important finding was that the age-standardized prevalence of clustering of ≥1, ≥2, and ≥3 CRFs in Chinese Kazakh, Uygur, Mongolian and Han populations was higher than subjects in the InterASIA Study (China) and close to their counterparts in the USA. This finding indicated that the clustering of CRFs is increasing at a rapid speed in China during recent years with the increasing prevalence of hypertension, DM, dyslipidemia, smoking, obesity and being overweight. Also, effective population-based interventions such as smoking cessation, improved diet (reduction of salt and fat), and increased physical activity can safely and effectively lower the risk of CVD [24,25]. A multifaceted and targeted approach aimed at prevention, detection, and treatment of hypertension, dyslipidemia, DM, and obesity could substantially reduce the prevalence of each CRF, CRF clustering, as well as morbidity and mortality from CVD in Xinjiang.

The strengths of the present study include the fact that its results are based on findings in a large, representative sample of adult Chinese Kazakh, Uygur, Mongolian and Han populations. In addition, a high response rate was achieved, standard protocols and instruments were used, the training and certification requirements for data collection were strict, and a vigorous quality-assurance program ensured that high-quality data were collected. Co-existence of different ethnic populations is common in many countries. Their genetic background, lifestyle, and environmental exposures have extensive impacts on risk factors and diseases. Elucidating the effects of these factors and adopting preventive measures will help to reduce the occurrence of diseases and improve health.

A limitation of the present study was reliance upon estimates derived from a cross-sectional study. Cross-sectional studies do not allow for quantification of the importance of CRF clustering in the prevalence of CVD. Nevertheless, a further prospective study could be considered among this population in the future. Moreover, physical inactivity was excluded as a CRF in the present analyses because it is causally involved in the development of all the CRFs investigated except for cigarette smoking. Inclusion of physical inactivity as a risk factor would have artificially increased the prevalence of CRF clustering. Finally, we recognize that diversity is present within each of the four ethnic groups studied, and that the ethnic differences presented in this study are the results of complex interactions between genetics, lifestyle, socio-economic status, provision of healthcare, and reporting. Further examination of these interactions is necessary.

## Conclusions

Despite universal access to healthcare, Chinese Uygur, Kazakh, Mongolian and Han groups living in Xinjiang differed markedly in their CRF clustering. These findings suggest that there may be a need to develop ethnic-specific and cost-effective strategies for preventing CVD in Xinjiang. These include strategies for preventing DM that are targeted specifically to high-risk Uygur populations, designing obesity-, hypertension- and dyslipidemia-prevention programs for Uygur, Kazakh and Mongolian populations, and promoting smoking prevention among Mongolian and Han populations. These ethnicity-tailored strategies might help to reduce ethnic disparities in CRFs and the burden of CVD.

## Competing interests

The authors declared that they have no competing interests.

## Authors’ contributions

NL conceived the study, participated in its design and coordination, and helped to draft the manuscript. HW participated in the design of the study and drafted the manuscript. ZY, XY, JH, LZ participated in the design of the study and undertook statistical analyses. All authors approved the final manuscript.

## Pre-publication history

The pre-publication history for this paper can be accessed here:

http://www.biomedcentral.com/1471-2458/12/499/prepub
